# Evaluating and rating HIV/AIDS mobile apps using the feature-based application rating method and mobile app rating scale

**DOI:** 10.1186/s12911-022-02029-8

**Published:** 2022-10-30

**Authors:** Ahmad Raeesi, Reza Khajouei, Leila Ahmadian

**Affiliations:** 1 Student Research Committee, Mashhad University of Medical Sciences, Mashhad, Iran; 2 Department of Health Information Sciences, Kerman University of Medical Sciences, Kerman, Iran; 3 Department of Health Information Sciences, Faculty of Management and Medical Information Sciences, Kerman University of Medical Sciences, Kerman, Iran; 4 HIV/STI Surveillance Research Center, WHO Collaborating Center for HIV Surveillance, Institute for Futures Studies in Health, Kerman University of Medical Sciences, Haft-Bagh Highway, Kerman, Iran

**Keywords:** HIV/AIDS, Mobile app rating scale, MARS, Feature-based application rating method, FARM

## Abstract

**Background:**

The purpose of this study was to evaluate HIV/AIDS mobile applications using the Mobile App Rating Scale (MARS) and rate the features of these applications using the new tool called the Feature-based Application Rating Method (FARM).

**Methods:**

In this study, all available HIV/AIDS apps in Iran from Cafe Bazaar and Google Play Store due to inclusion criteria were studied. The evaluation of the quality of applications was done using the MARS tool and the FARM tool. The FARM, which was developed in this study, was applied to rank the features of the applications.

**Results:**

In this study, 79 applications were included. The mean score of all apps using both tools was 3.58 (SD = 0.95) out of 5. The overall mean quality score based on the MARS was 3.14 (SD = 0.84), and the mean score of features based on FARM was 3.81 (SD = 1.23). This study showed a higher than moderate correlation between the scores assigned to apps based on the MARS and FARM tools (r > 0.4).

**Conclusions:**

The HIV/AIDS mobile applications available in Iran had the "acceptable" ranking. Also, our study results showed that to evaluate mobile apps, using a single tool may not provide good insight to evaluators about the assessed apps. However, using more than one tool may provide more details about the evaluated apps. To improve the quality of mobile health apps and help users select the most desirable app, we suggested using tools like FARM for ranking apps based on the features of each app in the app stores.

**Supplementary Information:**

The online version contains supplementary material available at 10.1186/s12911-022-02029-8.

## Background

The high penetration rate of mobile phones worldwide makes mobile applications one of the fastest-growing technologies [1]. Approximately 70% of mobile phones use the Android operating system [2]. During the second quarter of 2022, almost 3.5 million Android apps were available in the Google Play Store [3]. Of these, 54,603 are mobile health apps [4]. Mobile health apps have many uses in preventing and treating chronic diseases like HIV/AIDS [5–8]. HIV/AIDS is one of the most serious socio-economic threats to public health due to its chronic nature, the possibility of its prevalence among individuals, and the absence of a cure for this disease [9]. Approximately 38.4 million and 53 thousand people were living with HIV/AIDS in the world and Iran, respectively [10, 11].

Mobile applications related to HIV/AIDS can help communicate with care providers and reduce hospital care. These applications can be used to provide self-care and help patients achieve compliance with antiretroviral therapy. Moreover, these mobile applications can be used to send alerts and reminders, collect data, provide real-time audio and video communication, deliver educational information, and provide requested information to the community to prevent the disease and control its transmission to others [7, 12, 13]. The number of these apps and their features change over time [3, 14]. Despite the high number of mobile health apps and their features, the quality and validity of many apps are unknown [15, 16]. Therefore, there is a need for tools to continuously review and evaluate these apps to determine their quality and validity.

Several tools have been used to evaluate mobile apps [17–22]. These tools evaluate mobile apps based on quality and trustworthy health information [18, 19], some objective and subjective components [20], functionality scoring [21], and usability [22]. One of these tools is the Mobile Application Rating Scale (MARS), which evaluates and rates mobile apps in terms of qualitative, objective, and subjective aspects that were developed in the previous study by Stoyanov et al. [17]. The MARS tool consists of 23 questions in four objective dimensions (A_D), including Engagement (5 questions), Functionality (4 questions), Aesthetics (3 questions), Information Quality (7 questions), and a Subjective dimension (E) (4 questions) [17, 23]. The MARS tool is a comprehensive and reliable tool that is widely used to rate the quality of mobile health apps like Epilepsy, COVID-19, self-management mobile health apps, Spine disorders, and Alzheimer's disease [24–28]. This tool has been widely translated and validated into other languages, including French [29], Italian [30], Korean [16], Spanish [31], Japanese [32], Arabic [33], and German [34].

Although the MARS tool can evaluate various aspects of a mobile application, it has limitations in assessing the mobile application's features [35, 36]. In an app, a feature is typically an essential function or a service provided by the app for users [14]. Features may be desirable or undesirable to users. If the existence of a feature is positive and useful for the users, that is a desirable or positive feature. If the existence of a feature is negative for users, such as advertisements, or its presence is irritating for users, such as the presence of corrupted and misleading links, that is an undesirable or negative feature [37–39]. Some previous studies [35, 40–44] that used the MARS tool to evaluate the apps also reviewed the existence of features without reviewing the quality of each feature separately. The only tool that was developed to rate the mobile app features is the IQVIA functionality score (previously known as the IMS functionality score) [26]. This tool is based on seven functionality criteria and four functional subcategories detailed in the report of the IQVIA Institute for Healthcare Informatics [21, 45]. In some previous studies [26, 43, 45], this tool was used along with the MARS tool to rate mobile health app features. The IQVIA functionality score focuses on the availability of the 11 previously determined functionalities, and finally, each mobile app gives a score between 0 and 11. The MARS functionality score is an overall score on a five-point Likert scale that is measured based on the quality of performance, navigation, ease of use, and gestural design of that app [17, 21, 45]. The IQVIA evaluates the availability of functionality of each feature without considering quality, and the functionality section of the MARS tool evaluates the overall quality of functionality of each app. The functionality score range of IQVIA differs from the functionality score of MARS, so their scores are not comparable. The IQVIA feature lists are predetermined and not flexible for each app. Also, in these two tools, undesirable features are not considered.

Due to the high number of mobile apps in app stores, it is difficult for users to find their desired applications [46]. Currently, users choose a mobile application based on the application's popularity, star rating, comments on app stores, and the number of downloads, regardless of the quality of the application [47]. Identifying and rating the quality of mobile apps and their features can help users find and select an app based on the features they need [14, 37]. Mobile apps may have different features compared to each other. The previously developed evaluation tools [17–19, 21, 48, 49] have not adequately addressed the evaluation of each mobile app feature. When users are faced with an abundance of similar apps with many functionalities or features, they tend to choose apps with their desired features [14]. Therefore, it is necessary to develop a tool that has flexibility based on the availability of each feature on a certain app to evaluate and rate mobile apps.

Some mobile apps related to HIV/AIDS exist in app stores [50]. The literature review showed that few studies [50–53] evaluated the quality of HIV/AIDS-related mobile applications, and none of these studies had rated these applications' features. Despite the important role of mobile apps in HIV/AIDS prevention and treatment, no study has been conducted to review HIV/AIDS mobile apps in Iran. Therefore, the purpose of this study was to (1) evaluate the quality of HIV/AIDS-related mobile applications in the Google Play and Café Bazaar stores available in Iran using the Mobile Application Rating Scale (MARS); and (2) to evaluate and rate that applications' features (desirable and undesirable) using the new tool called the Feature-Based Application Rating Method (FARM).

## Methods

This article is the second part of a two-part series regarding evaluating HIV/AIDS-related applications in various terms, including their features and content [15]. This study was a cross-sectional descriptive-analytical study carried out on HIV/AIDS mobile applications in the Persian or English language available in Iran from May 6, 2021, to September 23, 2021. All HIV/AIDS-related mobile apps in the following two app stores were evaluated: The Google Play Store, the world's largest app store of mobile apps [3], and Café Bazaar, the most prominent Iranian app store for Persian mobile applications [54]. More than 3500 Android mobile applications in the Café Bazaar are related to the health and medical fields [55]. Given the small population size, all HIV/AIDS-related applications were included in the study.

Mobile apps are searched using the keywords "HIV", "AIDS", "Human Immunodeficiency Virus", "Acquired Immunodeficiency Syndrome", and the Persian keywords with similar meanings in the Google Play Store and Café Bazaar store. Then, the downloaded apps were installed on the Android smartphone (SAMSUNG Galaxy A51). The inclusion criteria to enter into this study are: (1) the mobile application can be installed on the Android operating system (Android 11.0); (2) it is written in Persian or English; (3) it is available in Iran, and (4) the focus of the mobile app is on HIV/AIDS.

Two evaluators with a background in health information technology independently evaluated all mobile apps using the MARS and FARM tools. Before evaluation, evaluators watched the MARS training video [56] and were trained about using the FARM tool. They are not the HIV/AIDS mobile app's real users. These two evaluators passed the related courses on the evaluation of mobile health apps. The first evaluator performed the evaluation after downloading and installing the included apps on the smartphone. When the evaluation was completed by the first evaluator, the stored data during the evaluation of the first evaluator inside of the apps was deleted, and then the second evaluator started the evaluation of the apps. Both evaluators first evaluated the HIV/AIDS apps using the MARS tool and then used the FARM. The evaluation sessions were limited to a maximum of 45 min per each session. We also asked the evaluators to spend sufficient time to gather the required information before assigning the scores. The evaluation was done when the evaluators were mentally prepared to perform the evaluation, and if the evaluators were tired, the evaluation stopped and continued to another time when the evaluators had sufficient mental preparation. The differences between the scores of the two evaluators were resolved by discussion between them. If the differences were not resolved through discussion between them, we used the third evaluator (supervisor) to resolve the discrepancy. The collected data was recorded on a paper form and then entered into a Microsoft Excel spreadsheet and SPSS for analysis.

In this study, all apps were downloaded directly from the Google Play Store and Cafe Bazaar. Our searches in the Google Play Store showed that all HIV/AIDS available mobile apps in Iran are free of charge. Also, the previous study [50] stated that all HIV/AIDS-related apps are available free of charge. In this study, the paid apps of Cafe Bazaar were included and evaluated.

### Data collection tools

The MARS and FARM tools were used to rate the mobile apps. This study was conducted to apply the FARM tool to evaluate the apps based on their features. In this study, we used the MARS tool to compare the results of the FARM with a previously developed tool and also to get a better view of the existing HIV/AIDS apps. Since the MARS and other previously developed mobile app evaluation tools [17–19, 21, 48, 49] have not adequately addressed the evaluation of mobile app features, to address this issue in this study, a tool called the Feature-Based App Rating Method (FARM) was developed to rate mobile apps based on their features. The FARM evaluates and rates mobile apps based on both the availability and quality of each feature. The items of this tool are not predetermined; they are flexible based on each app's features.

To develop the FARM tool, all HIV/AIDS-related mobile apps in the Google Play Store and the Café Bazaar that were included in this study were reviewed, and all of their features were extracted, and a list of these features was prepared. This list was considered as the desirable features for the FARM. The list of undesirable features was also prepared based on a previous study [38] and the opinions of four experts who had checked the validity of the FARM. The FARM is available in Additional file 1. In total, 33 desirable features and nine undesirable features were identified and added to the FARM. To determine a ranking method for the features of the mobile apps and create a ranking method in line with previously developed tools. The ranking methods of the previously developed tools [17–20, 45, 48, 57] were reviewed. In the FARM, we used the standard 5-point Likert ranking method to compare apps with other tools that used the 5-point ranking method to rank the apps (such as star rating and the MARS tool).

Two medical informatics specialists confirmed the ranking method used in the FARM tool. A score of zero was assigned to the app to rank the applications based on the FARM tool for the absence of each desirable feature. If the app contained a desirable feature, the evaluators checked its functionalities and assigned a score of one to five (1-inappropriate, 2-poor, 3-acceptable, 4-good, and 5-excellent) to that feature based on the extent to which the feature met its expected function. Moreover, to rank an undesirable feature, a score between one (the undesirable feature is very annoying) and five (the absence of the undesirable feature) was assigned to that feature.

The validity of MARS and FARM was confirmed by two Medical Informatics specialists and two Health Information Management specialists. To investigate the reliability of the MARS and FARM tools, the first 20 mobile apps retrieved from the Google Play Store were evaluated using these tools, and Cronbach's alpha was calculated for each tool. The internal reliability of the MARS tool was 0.94 for all questions. The internal reliability of the MARS dimensions was between 0.63 and 0.93. The internal reliability of the FARM tool was 0.85 for the desirable features and 0.76 for undesirable features.

### Data analysis

This study used descriptive statistics, including mean and standard deviation, to calculate the app ratings. To calculate the mean scores, the zero scores of the FARM tool and the N/A score of the MARS tool were not considered. The mean scores for the MARS and FARM tools were classified as the scores between 1 to 2 being considered as "inappropriate", 2 to 3 as "poor", 3 to 4 as "acceptable", 4 to 5 as "good", and 5 as "excellent". The Kolmogorov–Smirnov normalization test did not confirm the normality of variables related to the MARS and FARM tools. Therefore, the Spearman correlation test was used to examine the relationship between the dimensions of the MARS and FARM tools. The internal validity and consistency of the evaluators were calculated using the two-way mixed internal correlation coefficient (ICC) [58]. Microsoft Excel version 2019 was used to analyze the descriptive data, and SPSS version 24 was used to analyze the analytical statistics.

## Results

**Fig. 1:**
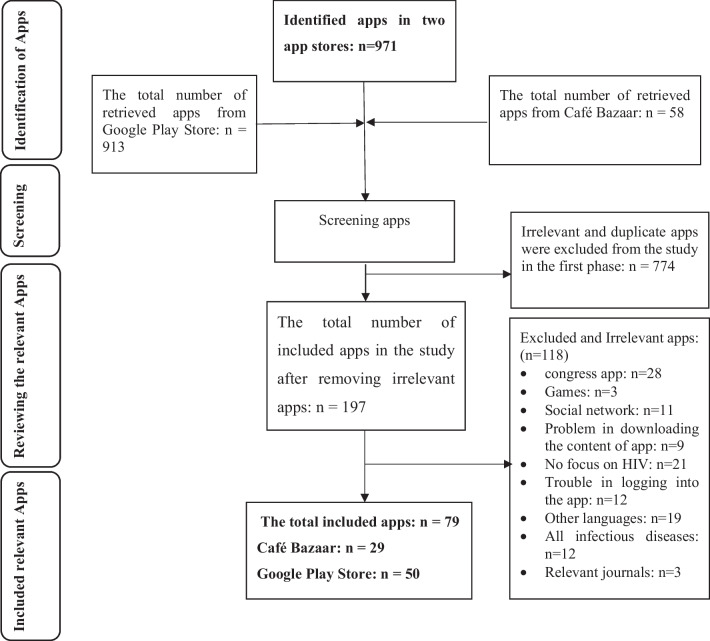
Flow chart of the selection process for inclusion of the Apps

A total of 971 apps were retrieved from the two app stores, of which 79 apps based on the inclusion criteria were included in the study. Of these, 29 apps were in the Café Bazaar and 50 were in the Google Play Store. All HIV/AIDS apps available in the Google Play Store are free. Five of 29 (17%) Café Bazaar applications are paid apps, and 14 (48%) are in-app purchases. The process utilized to identify the apps is shown in Fig. 1.

The mean rating score of apps in stores was 4.37 (SD = 0.60). The organizational affiliations of 19 apps (38%) in the Google Play Store were unknown; one app (2%) was commercial, 14 apps (28%) were governmental, non-governmental organizations developed ten apps (20%), and the affiliations of 6 apps (12%) were universities. The organizational affiliations of 13 apps (45%) in the Café Bazaar were unknown; thirteen apps (45%) were commercial; three apps (7%) were non-governmental organizations, and the university developed one app (3%).

### The results of the evaluation of the mobile app

The results of evaluating HIV/AIDS-related apps using the MARS and FARM tools are shown in Table 1. The average score of all apps using both tools was 3.53 (SD = 0.68) out of 5. As Table 1 shows, the overall mean score for Google Play Store apps was 3.74 (SD = 0.68), and for Café Bazaar apps it was 3.15 (SD = 0.52). Among all the dimensions of both tools, "undesirable features" was the highest-scoring domain (4.67 ± 0.42). The lowest score was related to "Engagement" (2.85 ± 0.93).

**Table 1:** The results of the evaluation of HIV/AIDS apps based on MARS and FARM tools

	FARM dimensions		FARM total score	MARS dimensions					MARS total score	Total score
	Desirable Features	Absence of undesirable feature		Engagement	Functionality	Aesthetics	Information Quality	Subjective dimension		
	Mean (SD)	Mean (SD)	Mean (SD)	Mean (SD)	Mean (SD)	Mean (SD)	Mean (SD)	Mean (SD)	Mean (SD)	Mean (SD)
Cafe Bazaar apps	2.53 (0.74)	4.60 (0.32)	3.59 (0.47)	2.64 (0.67)	3.99 (0.47)	3.10 (0.98)	2.32 (0.74)	1.80 (0.73)	2.71 (0.62)	3.15 (0.52)
Google Play Store apps	3.22 (1.10)	4.71 (0.47)	4.12 (0.73)	2.98 (1.04)	4.01 (0.66)	3.38 (0.81)	3.55 (0.97)	2.92 (1.32)	3.37 (0.85)	3.74 (0.68)
Mean of all apps	2.93 (1.02)	4.67 (0.42)	3.92 (0.69)	2.85 (0.93)	4.00 (0.59)	3.28 (0.88)	3.10 (1.07)	2.50 (1.26)	3.13 (0.83)	3.53 (0.68)

Fourteen apps (28%) out of 50 apps in the Google Play Store give a score above 4: "Every Dose, Every Day" (4.76 ± 0.29), "HIV Oral PrEP Implementation Tool" (4.75 ± 0.40), "inPractice HIV" (4.71 ± 0.30), "HIV Client Treatment Preparedness" (4.70 ± 0.24), "HIV Care Tools" (4.65 ± 0.30), "WHO HIV Tx" (4.59 ± 0.38), "Liverpool HIV iChart" (4.55 ± 0.27), "YourPrEP" (4.53 ± 0.34), "WHO HTS Info"‏ (4.51 ± 0.39), "HIV-HCV Drug Therapy Guide" (4.51 ± 0.39), "ClinicalInfo HIV/AIDS Guidelines" (4.45 ± 0.40), "life4me + " (4.24 ± 0.53), "EACS" (4.08 ± 0.79), and "HIV-Rx DDI Check" (4.02 ± 0.66). Also, two apps (7%) out of 29 Cafe Bazaar apps give scores above 4: "Pishgiriye Pas az Tamas (PEP)" (4.49 ± 0.44), and "Agahi Bakshi AIDS" (4.18 ± 0.60). The lowest score among all apps was taken by the Cafe Bazaar app named "AIDS va Darman" (2.02 ± 1.31). Among all of the Google Play Store HIV/AIDS rated apps, 14 apps (28%) scored above 4, 23 apps (46%) scored between 3 and 4, and 13 apps (26%) scored between 2 and 3. Among all of the Cafe Bazaar HIV/AIDS rated apps, two apps (7%) scored above 4, nine apps (31%) scored 3 to 4, and 18 apps (62%) scored between 2 and 3.

### The MARS tool results

The MARS total mean score for all apps was 3.13 ± 0.83. The highest rank was related to the Functionality dimension (4.00 ± 0.59), and the lowest was related to the Subjective dimension (2.50 ± 1.26) (Table 1). Fifteen apps (19%) scored above 4 out of 5. The highest three scored apps were "HIV Oral PrEP Implementation Tool" (4.82 ± 0.66), "inPractice HIV" (4.74 ± 0.45), and "Every Dose, Every Day" (4.71 ± 0.56). Also, 23 apps (29%) scored 3, 38 apps (48%) scored 2, and only three apps (4%) scored 1. The lowest three scored apps were "HIV AIDS Awareness" (1.77 ± 0.92), "AIDS va Darman" (1.80 ± 1.06), and "AIDS va Moghabele ba an" (1.86 ± 0.96).

### The results of ranking mobile app features using the FARM tool

The results of the ranking of HIV/AIDS mobile app features using the FARM tool are shown in Table 1. The FARM mean score of all apps was (3.92 ± 0.69) out of 5. The apps retrieved from Cafe Bazaar did not have 21 (63%) of the 33 evaluated features in this study. All the Cafe Bazaar apps had a textual content. The features "app description inside of the app" (3.50 ± 1.29), the "search functionality" (3.16 ± 0.71), and the "Bookmark feature" (3.12 ± 0.83), respectively, scored the highest rank.

The ranking of the Cafe Bazaar HIV/AIDS apps based on undesirable features showed that features such as "being a free app but requiring payment for basic features" (3.86 ± 1.16), "advertising" (4.00 ± 0.80), and "the existence of unrelated information" (4.28 ± 0.84) had the lowest score for Café Bazaar apps. The features "Difficulties to login into the app" (5.0 ± 0), "Inactive and misleading buttons" (4.97 ± 0.19), and "stopping the app after execution" (4.97 ± 0.19) had the highest ranking.

In Google Play Store apps, evaluators assigned the highest scores to the features "Collect medication data" (4.37 ± 0.51), "medication management and medication reminder" (4.28 ± 1.49), and "documentation and presentation of the disease progression" (4.00 ± 1.73). The lowest scores were assigned to desirable features such as "direct interaction and visual contact" (1.14 ± 0.35), "direct audio contact" (1.38 ± 0.49), and "communication with people with similar conditions" (1.40 ± 0.49).

According to the ranking results of the Google Play Store apps based on the absence of undesirable features, the lowest scores were assigned to "corrupted and misleading links" (4.50 ± 0.91), "inactive and misleading buttons" (4.60 ± 0.78), and "taking a long time to load the content of the app after executing the program" (4.66 ± 0.89). The highest ratings in this regard were related to "being a free app but requiring payment for basic features" (4.88 ± 0.44), "difficulties in logging into the app" (4.84 ± 0.51), and "advertising" (4.82 ± 0.48).

The agreement rate between the two evaluators regarding the app's rating based on FARM and MARS tools is shown in Table 2. The agreement rate between the two evaluators for the overall MARS score, calculated using the ICC, was 0.947 (CI 95% = 0.919–0.965). The agreement between the two evaluators for the overall FARM score was 0.882 (CI 95% = 0.819–0.922).

**Table 2:** The agreement rate between the two evaluators regarding the rating based on FARM and MARS

Tools	Dimensions	ICC	Confidence interval of 95% for ICC
MARS	Engagement	0.916	0.871–0.945
	Functionality	0.930	0.893–0.954
	Aesthetics	0.840	0.756–0.895
	Information quality	0.944	0.913–0.964
	Subjective dimension	0.919	0.866–0.950
FARM	Desirable features	0.931	0.892–0.955
	Absence of undesirable feature	0.537	0.296–0.696

The relationship between the dimensions of the MARS tool and the FARM tool was calculated using the Spearman correlation test, and the results are shown in Table 3. The lowest correlation was found between the score of the Subjective dimension and the functionality score of the MARS tool (r = 0.450). The highest correlation was found between the score of the Subjective dimension and the Information quality score of the MARS tool (r = 0.832).

**Table 3:** The correlation between the FARM dimensions and The MARS dimensions

Tools and dimensions		FARM dimensions		MARS dimensions				
		Desirable Features	Absence of	Engagement	Functionality	Aesthetics	Information quality	Subjective dimension
FARM dimensions	Desirable feature	1.0	0.574	0.734	0.461	0.615	0.753	0.745
	Absence of undesirable features	0.574	1.0	0.555	0.499	0.514	0.538	0.524
MARS dimensions	Engagement	0.734	0.555	1.0	0.620	0.713	0.684	0.802
	Functionality	0.461	0.499	0.620	1.0	0.603	0.464	0.450
	Aesthetics	0.615	0.514	0.713	0.603	1.0	0.660	0.643
	Information quality	0.753	0.538	0.684	0.464	0.660	1.0	0.832
	Subjective dimension	0.745	0.524	0.802	0.450	0.643	0.832	1.0

## Discussion

The results of this study showed that there is a higher than the moderate correlation between the scores assigned to apps based on the MARS and FARM tools. Therefore, the high MARS score somewhat indicates the existence of desirable and the absence of undesirable features in the app. So, evaluating mobile apps by using a single tool may not provide good insight about the evaluated apps. However, using more than one tool provides more details about the evaluated apps.

According to the results of this study, the HIV/AIDS mobile applications available in Iran had an "acceptable" ranking. However, the overall ranking score of the MARS tool and desired features for Cafe Bazaar apps scored "poor". In previous studies [27, 44, 59–62] conducted on health applications using the MARS tool, evaluated apps were ranked as "acceptable". Moreover, the results of the study done by Young et al. [63] showed that the overall quality of the apps for men who have sex with men (MSM) in China is "acceptable". In this study, we evaluated all HIV/AIDS mobile applications available in Iran, including mobile applications related to HIV pre-exposure prophylaxis. Sharpe et al. [64], in 2018, only evaluated 11 mobile apps for HIV pre-exposure prophylaxis using the MARS tool. Just one mobile app (PreP4U) reviewed in the study by Sharpe et al. [64] was available in Iran in 2021 and was also reviewed in our study. The results of the average ratings of this mobile app were almost the same as in our study.

The FARM does not check the quality of information. In this study, the quality of information is checked with the "information quality" section of the MARS tool. The Google Play Store apps were ranked "acceptable" in terms of "information quality", but the Cafe Bazaar apps were rated "poor". The first published part of our study rated the HIV/AIDS mobile apps based on the evidence showed that the Cafe Bazaar apps were rated as "inappropriate" and the Google Play Store applications were rated as "good" [15]. Due to the importance of the information content of an app [65], it is necessary to evaluate and, if possible, eliminate apps with inappropriate information from app stores. The results of our study confirm the results of the Robustillo Cortés et al. [53] study conducted in 2013. Their study indicates that the quality of the evaluated apps on HIV is limited, and only one app (inPractice HIV) is categorized in Class A. This app, in our study, was ranked as "good". According to the results of our study, of all the Café-Bazaar apps, only one has been written by health organizations. Also, the affiliation of more than half of the apps was not determined, and they may not be reliable. This is in line with the results of a study by Rosa et al. [52], which showed that more than half of the apps were not written by health professionals.

In this study, the mean FARM score for all desirable features was low. Most apps were ranked with a low score in terms of their desirable features. However, the high number of features considered in the FARM tool may affect the total desirable score results. Also, the Functionality dimension of the MARS tool had the highest score among all other dimensions of the MARS tool. According to the Functionality dimension, all apps ranked higher than the "acceptable" score, but most ranked as "inadequate" in terms of desirable features. In most previous studies using the MARS tool [26, 28, 30, 60, 66], the functionality score was the highest compared to the scores of the other dimensions of the MARS tool. Therefore, ranking apps with the Functionality dimension of the MARS tool and its general questions cannot conclude that the apps contain desirable features. In the study of Schnall et al. [50], only nine features of HIV/AIDS-related mobile apps were evaluated, and the results had not been compared with other valid tools, nor had the evaluated features been ranked. Our study confirmed the results of the Schnall et al. study [50], which showed that a small number of HIV/AIDS-related apps have the desired functionalities.

In this study, the best features of the HIV/AIDS mobile apps were collecting medication data, medication management and medication reminders, and documentation and presentation of the disease progression. A previous study [67] showed that the most interesting features for HIV/AIDS patients' needs are the reminders/alerts feature, collecting lab data and lab results tracking, and notes about health status. These features were better designed than other features, but they need to be given more attention and designed better.

Based on our results, the lowest undesirable feature score of the Cafe Bazaar apps was assigned to the "being a free app, but requiring payment for basic features" and "advertising" features. The Cafe Bazaar apps did not have a good status in terms of the existence of undesirable features. Although an app may have rich content, it's very annoying for users to face a lot of advertising and unwanted features.

The FARM evaluates and rates mobile apps based on the availability and quality of each feature. The items of the FARM are flexible based on each app's features. In this study, we included all the features available at the time of the study. The features of the apps may change with each update, and apps may contain different features compared to each other. Feature lists also differ from one disease to another. The list of FARM features may change over time with app updates and with a decrease or increase in the number of included apps. The FARM has flexibility and can be used to rank mobile apps to help users choose the app they want.

Currently, most of the features of mobile apps in the App Store are unknown to users, a few apps have mentioned the features in the app description section, but the quality of these features is unknown. So, the results of evaluated HIV/AIDS mobile apps in this study can be helpful for these app users to choose their desired apps. The desirable and undesirable features extracted in the FARM can help mobile app developers to develop new applications for HIV/AIDS patients. Furthermore, the tools used in this study to rate the desirable and undesirable features of mobile apps can be used by researchers to evaluate mobile apps in future works. Further research is required to investigate the implications of the FARM tool.

For future work, we recommend mobile app evaluators use and test the FARM tool to evaluate other mobile health apps. For studies that use the FARM, it is suggested to first prepare a list of the features of the mobile apps that they want to evaluate, then rank the quality of each feature of a mobile app using the FARM, and finally, the mean of these scores is the mobile app's overall score. The FARM can be used for just one app. Mobile app developers can use the FARM to rate their mobile apps. It is also suggested to divide the features into sub-features, first assign a score to the sub-features, and then calculate the feature score based on the average score of the features. Moreover, we recommend using quantitative methods like quantitative usability methods for each feature instead of qualitative methods. Also, for qualitative evaluations, use more than two evaluators or real users.

## Strengths and limitations of the study

Using the five-choice ranking in the developed tool in this study for rating app features is one of the strengths of this study because this made the results comparable with the results of the evaluations done with the other tools with the same ranking [17, 19]. Moreover, in this study, we ranked the desirable and undesirable features available in the apps and compared the rankings with the MARS tool.

This study has several limitations. First, this study was conducted on mobile applications available in Iran. Since not all applications are available in Iran due to the sanctions against the country and regional restrictions, this study was conducted in a country with limited access to mobile apps, and therefore the results may not be generalizable to other HIV/AIDS-related mobile apps available in other countries. However, in terms of the number of evaluated HIV/AIDS apps, this study has the highest number of apps being assessed compared to the previous studies [50–53].

Second, in this study, mobile apps running on the IOS operating system were not evaluated due to sanctions against the country and the removal of Iranian apps from this store. To deal with this issue, the researcher wrote to Apple Company to access the mobile apps for conducting this research but failed to obtain the necessary permission. According to the findings of a previous study [50], more than 60% of HIV/AIDS mobile apps are available on the IOS and Android platforms.

Third, another limitation of this study was using two evaluators to rate 79 mobile apps with many features using FARM and MARS. We used two evaluators because most of the previous studies that used the MARS tool for evaluating mobile health apps used two evaluators [16, 17, 29–33, 36, 42, 44, 45, 64]. The evaluation process may be affected by evaluator bias, fatigue bias, experience bias, and familiarity bias with using two evaluators. To reduce the above mentioned biases in this study, the evaluators were trained about biases before conducting the evaluation. The evaluation was performed when the evaluators were mentally prepared to complete the evaluation, and if the evaluators were tired, the evaluation was stopped and continued until another time when the evaluators had sufficient mental preparation.

Fourth, according to similar studies [15–20, 26, 35], we applied a subjective approach to evaluate HIV/AIDS mobile apps. Given to limitations of subjective evaluation methods, using quantitative methods is recommended for future studies.

## Conclusions

In this study, we used the MARS tool and a new tool called the FARM to evaluate desirable and undesirable features of HIV/AIDS mobile apps. This study showed that the rank of HIV/AIDS-related available apps in Iran is "acceptable". According to the results of the MARS tool and desirable features, Cafe Bazaar apps were ranked as "poor" and lacked a third of the desirable features. The developers of the Cafe Bazaar apps should add some features based on users’ needs to their apps.

The FARM can determine the desirable and undesirable features of mobile apps and the quality of those features and then rank mobile apps based on their features. Our study results also showed that using a single tool to evaluate mobile apps may not provide good insight to evaluators about the assessed apps. However, using more than one tool provides more details about the evaluated apps. The FARM is a new tool. Therefore, further studies are needed to test the FARM on mobile health apps in different health domains.

## Supplementary Information

**Additional file 1:** The Feature-Based Application Rating Method (the FARM) Tool.

## Data Availability

Data sharing is not applicable to this article as no datasets were generated or analysed during the current study.

## Abbreviations

FARM: Feature-based Application Rating Method
MARS: Mobile Apps Rating Scale
HIV: Human Immunodeficiency Virus
AIDS: Acquired Immune Deficiency Syndrome
ICC: Internal Correlation Coefficient
SD: Standard Deviation
WHO: World Health Organization
CI: Confidence Interval
IOS: IPhone Operating System
