# Supplementary material for: Evaluating and rating HIV/AIDS mobile apps using the feature-based application rating method and mobile app rating scale
Source: BMC Med Inform Decis Mak. 2022 Oct 30;22:281. doi: 10.1186/s12911-022-02029-8 (PMC9618024; doi:10.1186/s12911-022-02029-8)
Supplement: Supplementary file 1 — Additional file 1: The Feature-Based Application Rating Method (the FARM) Tool. [file 12911_2022_2029_MOESM1_ESM.docx]

The Feature-Based Application Rating Method (the FARM)

# Section 1: Apps Information

**App Name**: _________________________________________________

**Developer**: _________________________________________________

**Rate of the Apps in the store**: _________________________________

**Number of reviewer in the store**: _______________________________

**Version**: ______________________________ **Last update**: ______________

**Platform**: IOS Android

**Store name**: Café Bazaar Google Play Store Apple App Store

**Affiliations**: Unknown Commercial Government NGO Academic

**Brief description**: ________________________

# Section 2: Desirable features

The evaluator assigned a score of zero to the apps for the absence of desirable features. If the app had a desirable feature, the evaluators checked how its functionalities worked and assigned a score of one (weak) to five (excellent) to that feature based on the extent to which the feature met its expected function.

| Row | Desirable features | | Don’t exist=0 | Exist=1 to 5 | |
| --- | --- | --- | --- | --- | --- |
|  | Collect patient data | Collect medication data |  |  | |
|  |  | Collect lab result data |  |  | |
|  |  | Collect changes in emotions and mood data |  |  | |
|  |  | Collect demographic data, e.g. Age, gender |  |  | |
|  |  | Other collected data: … |  |  | |
|  | Communication feature | Communication with the health care providers |  |  | |
|  |  | Communication with people with similar conditions |  |  | |
|  |  | Communication with the app developers (Contact Us) |  |  | |
|  | Communication ways | Direct video contact |  |  | |
|  |  | Direct voice contact |  |  | |
|  |  | Ask questions and answer (text) |  |  | |
|  |  | Voice, video, and text communication (all above communication ways) |  |  | |
|  | Medication management | Medication interaction |  |  | |
|  |  | Medication reminder |  |  | |
|  |  | Medication alert |  |  | |
|  | Help feature | |  |  | |
|  | Note feature | |  |  | |
|  | Search feature | |  |  | |
|  | Bookmark feature | |  |  | |
|  | The ability to share the app's content | |  |  | |
|  | Password and encryption features | |  |  | |
|  | Data import and export features | |  |  | |
|  | Links to additional information inside the app | |  |  | |
|  | References list | |  |  | |
|  | Ability to present content (information) based on patient information | |  |  | |
|  | Documentation and presentation of the disease progression | |  |  | |
|  | App description inside of the app | |  |  | |
|  | Setting feature | |  |  | |
|  | Calendar feature | |  |  | |
|  | Find the nearest healthcare provider | |  |  | |
|  | GPS functionality | |  |  | |
|  | Feasibility of updating the content of the app | |  |  | |
|  | Other desirable features: ……… | |  |  | |
| **Section 3: Undesired features** To rank an undesirable feature: a score between one (the undesirable feature is very annoying) and five (the absence of the undesirable feature) was assigned to that feature. | | | | | |
| Row | **Undesirable features** | | **Don’t exist=5** | | **Exist=1 to 5** |
|  | Advertisement | |  |  | |
|  | Corrupted and misleading links | |  |  | |
|  | Inactive and misleading buttons | |  |  | |
|  | The existence of unrelated information | |  |  | |
|  | The app hangs after execution | |  |  | |
|  | It was a free app but required payment for basic features | |  |  | |
|  | Difficulties in logging into the app | |  |  | |
|  | It takes a long time to load the content of the app after execution | |  |  | |
|  | Other undesirable features: ……… | |  |  | |

**­­­­­­­­­­­­­­­**
